# Automated tracking of gene expression in individual cells and cell compartments

**DOI:** 10.1098/rsif.2006.0137

**Published:** 2006-06-14

**Authors:** Hailin Shen, Glyn Nelson, David E Nelson, Stephnie Kennedy, David G Spiller, Tony Griffiths, Norman Paton, Stephen G Oliver, Michael R.H White, Douglas B Kell

**Affiliations:** 1 School of Chemistry, The University of ManchesterFaraday Building, Sackville Street, PO Box 88, Manchester M60 1QD, UK; 2 Manchester Centre for Integrative Systems Biology, Manchester Interdisciplinary Biocentre, The University of Manchester131 Princess Street, Manchester M1 7DN, UK; 3 Centre for Cell Imaging, School of Biological Sciences, University of LiverpoolBiosciences Building, Crown Street, Liverpool L69 7ZB, UK; 4 School of Computer Science, The University of ManchesterOxford Road, Manchester M13 9PL, UK; 5 Faculty of Life Sciences, The University of ManchesterMichael Smith Building, Oxford Road, Manchester M13 9PT, UK

**Keywords:** high-content screening, image analysis, image processing, single-cell analysis, NF-κB signalling

## Abstract

Many intracellular signal transduction processes involve the reversible translocation from the cytoplasm to the nucleus of transcription factors. The advent of fluorescently tagged protein derivatives has revolutionized cell biology, such that it is now possible to follow the location of such protein molecules in individual cells in real time. However, the quantitative analysis of the location of such proteins in microscopic images is very time consuming. We describe CellTracker, a software tool designed for the automated measurement of the cellular location and intensity of fluorescently tagged proteins. CellTracker runs in the MS Windows environment, is freely available (at http://www.dbkgroup.org/celltracker/), and combines automated cell tracking methods with powerful image-processing algorithms that are optimized for these applications. When tested in an application involving the nuclear transcription factor NF-κB, CellTracker is competitive in accuracy with the manual human analysis of such images but is more than 20 times faster, even on a small task where human fatigue is not an issue. This will lead to substantial benefits for time-lapse-based high-content screening.

## 1. Introduction

There is increasing interest, in both cell biology and drug discovery, in knowing both the amount and the spatial distribution of specific proteins in individual cells. Aided by the development of luciferase tags and fluorescent proteins (e.g. Tsien 1998), optical methods can now be used to effect this, leading to a huge increase in cell-based or so-called ‘high-content’ screening assays (Dove 2003; Abraham *et al*. 2004; Carpenter & Sabatini 2004; Giuliano *et al*. 2005; Grånäs *et al*. 2005; Mitchison 2005; Bailey *et al*. 2006). Many of these assays are currently performed at fixed time points, whereas it is becoming clear that for systems biology modelling it is important to track cellular functions in single cells over time. A major limitation for this has been the lack of analysis tools for time-lapse imaging in single cells. While the scoring of such image-based assays can (of course) be done manually, the volumes and complexity of the data generated make the development of automated scoring procedures highly desirable.

A number of commercial software systems offer general-purpose image-processing capabilities, while some of the commercial integrated hardware systems, based on automated microscopy, incorporate software that is designed to form part of specific assay kits, often in fixed cells. However, as part of a programme in understanding spatial signal transduction using live-cell imaging in single cells (e.g. Nelson *et al*. 2002*a*,*b*, 2003, 2004), it became clear that none of these was suitable for our needs, more specifically because living cells are motile and change shape throughout the course of time-lapse experiments. In addition, we wished to have a robust data model that would allow us to store and retrieve the images in a structured, effective, intelligent and systematic manner, much as in the emerging standard for the open microscopy environment (Swedlow *et al*. 2003; Goldberg *et al*. 2005).

Carpenter, Sabatini and colleagues have developed open source software for cellular image processing (e.g. Carpenter & Sabatini 2004; Wheeler *et al*. 2004; Bailey *et al*. 2006 and www.cellprofiler.org/), but it is not designed for tracking moving (living) cells. We have therefore developed, and here describe, CellTracker—an image-processing environment designed for the analysis of high-content cellular images.

## 2. Methods

Compared with most tracking tasks, CellTracker tracks not only cell positions, but also their boundaries. With cell boundaries available, one may measure gene expression level during dynamical processes as well as other cellular properties, e.g. morphology. There are three types of boundaries in the CellTracker, i.e. cellular, nuclear and user-defined. The user-defined boundaries can be used for tracking cellular compartments other than the cytoplasm and the nucleus. Boundaries are defined as two-dimensional cubic spline curves with the properties given in table 1. Only the control points of boundaries are usually used in the tracking, which also makes boundary editing easy. CellTracker provides management tools for boundaries over a time-series, e.g. copy, edit, delete, rename and conversion. It also includes operators for simple boundary processing activities, such as expansion and contraction.

**Figure 1:**
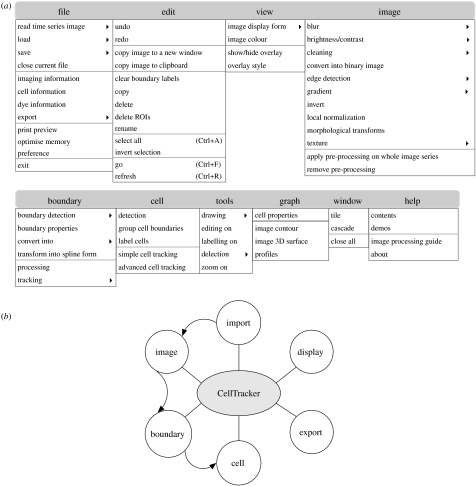
The basic functionality of CellTracker. (*a*) Illustration of the menu structure. (*b*) Logical flow of operations.

The menus and logical structure of CellTracker are illustrated in figure 1. CellTracker offers various visualizing tools. An image series may be inspected using various combinations of imaging channels. Boundaries are plotted as overlays upon image. Operations in CellTracker can be classified at the levels of the image, boundary or cell. Image processing and cell tracking are confined to user-selected regions of interest that persist over a sequence of images.

### 2.1 Image processing

Compared with natural images, image intensities inside cells are often not homogeneous and there can be large differences between the cells in a single image. As can be seen from figure 1, a number of image-processing techniques have been included, e.g. image blurring, gradient, edge detection, morphological transforms, local normalization and texture feature operations. Preview windows are usually given to help users to choose proper parameters. CellTracker also provides an image-processing guide under the help menu.

### 2.2 Boundary detection

Since CellTracker uses the fluorescent channels for object detection, the cells themselves may be detected based on image intensities, e.g. via thresholding and level set (Vese & Chan 2002). If an initial boundary is given, the CellTracker may refine the cell boundary based on the detected edges or absolute intensities. The active contour (‘snakes’) is an algorithm based on edge information (Kass *et al*. 1987), and is a curve χ(r)=[x(r),y(r)] that moves within an image to minimize the energy function

(2.1) $$E=\int_{0}^{1}(\alpha |\chi^{\prime }(r){|}^{2}+\beta |\chi^{\prime \prime }(r){|}^{2}+E_{\text{ext}}(\chi (r)))\text{d}r,$$

where *α*, *β* specify the elasticity and stiffness (respectively) of the active contour. The external energy function *E*_ext_ is derived from the image so that it takes on its smallest values at the features of interest, such as boundaries. There are quite a few formulations of the active contour. We use the algorithm developed by Xu & Prince (1998), where the external force is defined as the gradient vector flow (GVF) field. The main advantages of GVF active contours are: (i) a longer capture range to guide the contour towards the desired boundary, and (ii) an ability to progress into boundary concavities. The latter is very useful since cell boundaries may have sharp corners.

A Voronoi-based segmentation method is also used to find cytoplasmic regions (Jones *et al*. 2005). Given seeds for a region, the segmentation process is guided by the appearance of the cells. A metric is defined as

(2.2) $$G=\frac{\nabla g(F)\nabla g^{\text{T}}(F)+\lambda I}{1+\lambda },$$

where *F* is the image, *g* is a blurring filter with a small radius and *I* is the 2×2 identity matrix. *λ* is a regularization parameter, which makes the metric more Euclidean as it increases. Given the metric above, each foreground pixel is assigned to the nearest seed within the manifold defined by the metric. Boundaries between regions are specified where adjacent pixels are assigned to different seeds.

### 2.3 Boundary tracking

#### 2.3.1 Shape model

In many tracking applications, the object shape is modelled using a two-dimensional planar affine transform (Blake & Isard 1998) with only one shape template. In most cases, the nuclear boundaries undergo limited changes, which may also be modelled using the conventional approach. Any allowed shape vector ***Q*** can be represented by a shape space ***W***.

(2.3) Q=Ws.

Here, ***s*** is a vector for estimating ***Q*** within the shape space defined by ***W***. The shape matrix for affine transforms can be written as

(2.4) $$W=\left[\begin{array}{cccccc} 1 & 0 & Q^{x} & 0 & Q^{y} & 0\\ 0 & 1 & 0 & Q^{y} & 0 & Q^{x} \end{array}\right].$$

Here, **1** and **0** in equation (2.4) are vectors with 1s and 0s, respectively. *Q*^*x*^ and *Q*^*y*^ are the *x* and *y* coordinates of shape template on the key frames. The first two columns of ***W*** govern horizontal and vertical translations, respectively. *Q*^*x*^ and *Q*^*y*^ are chosen to have their centroids at the origin so that the third and thereafter columns are associated with shape changes only. The nuclear boundary can usually be represented by equation (2.4). In some cases, the shape of the nuclei approximates to an ellipsoid. The tracking parameter ***s*** contains the centre, radii and orientation of the ellipse. The affine shape variation is true only for a very limited number of cellular boundaries. They may be better approximated by a linear combination of those in the key frames, and the system thus uses a couple of representative boundaries as templates. A motion of translation and a linear combination of key frames 1 and 2 can be written as

(2.5) $$W=\left[\begin{array}{cccc} 1 & 0 & Q_{1}^{x} & Q_{2}^{x}\\ 0 & 1 & Q_{1}^{y} & Q_{2}^{y} \end{array}\right].$$

If the cell boundaries cannot be represented by a limited number of templates, the Voronoi-based boundary detection method (described in §2.2) may be employed, using the nuclear boundaries as seeds.

#### 2.3.2 Tracking features

Given a contour, the possibility of its being aligned with cellular boundaries may be estimated based on the appearance, edge or colour features. Correlation-based tracking is a traditional approach based on object appearance. It is incapable of tracking boundaries with varying shape and size. In edge-based tracking, an observation is made normal to a set of points chosen to lie on a contour; here, we used evenly spaced points. In the tracking algorithm, we sample a series of random contours in the image. Given edges detected along a normal of the contour, the probability of a sample reflecting a true contour point is estimated as follows (Isard & Blake 1998):

(2.6) $$p\propto 1+\frac{1}{\sqrt{2\pi }\sigma \lambda }\sum_{m=1}^{M}{\text{e}}^{-(f(d_{m},\mu )/2\sigma^{2})}\;\text{where}\;f(d_{m},\mu )=\text{min}(d_{m}^{2},\mu^{2}).$$

The parameter *σ* is similar to the standard deviation in a normal distribution and is set according to the accuracy of the shape model. The more accurate a shape model, the smaller is *σ*. *λ* is related to the prior probability of the contour point not being detected by edge detection. *d*_*m*_ is the distance between the contour and the edge along its *m*th normal. *μ* is the maximum distance between the contour and edge points under consideration. The probability of a hypothetical contour aligning with the true contour is estimated by multiplying the probabilities of edges along all the normal lines. If there is no edge detected, as we can seen from equation (2.6), $f(d_{m},\mu )=\mu^{2}$.

In colour-based tracking (Nummiaro *et al*. 2003), the colour template is characterized by the colour histogram in a region. The similarity between the colour distributions is measured by the Bhattacharyya distance *d* (see appendix). The observation probability of each sample is specified by a Gaussian with standard variation *σ* (equation (2.7)). More details about colour template tracking can be found in the appendix.

(2.7) $$\pi^{\left(n\right)}=\frac{1}{\sqrt{2\pi }\sigma }{\text{e}}^{-(d^{2}/2\sigma^{2})}.$$

#### 2.3.3 Dynamic model

The dynamics of shape parameters can be presented using an autoregressive model of order *K*. In equation (2.8), *s*_*n*_, and *s*_*n−k*_ are shape parameter vectors at times *n* and *n−k*, respectively. In the case of modelling in equation (2.4), the parameters involve *x*, *y* translation, shape scale and rotation. In an unsupervised tracking, ***A*** is set to the identity matrix, i.e. cell motion is regarded as random walk. A large noise level $\epsilon_{n}$ at time *n* has to be set in order to cover the possible range of motion. In the CellTracker, all the shape space contains *x*, *y* position parameters. With a training set, their corresponding dynamic parameters are determined by stepwise least squares (Schneider & Neumaier 2001).

(2.8) $$s_{n}=\sum_{k=1}^{K}A_{k}s_{n-k}+\epsilon_{n}.$$

For tracking using key frames, we assume that the cell boundaries vary steadily over time. The shape changes between frames, Δs, may be calculated beforehand. A small amount of random shape variation is added as well because the shape change is not necessarily evenly distributed between the key frames.

#### 2.3.4 Particle filter

The objective of tracking is recursive estimation of the boundary position and size *s*_*n*_ of the filtering distribution given a series of observations $y_{1:n}=(y_{1},\ldots ,y_{n})$. In the framework of probability theory, the objective is to estimate the conditional probability of *s*_*n*_, i.e. $p(s_{n}|y_{1:n})$. Generally, there are no analytical solutions for the above formulation. The particle filter (Doucet *et al*. 2001) is a Monte Carlo implementation of general recursions, in which the filtering distribution is represented by samples/particles with associated important weights ${\bar{p}}_{N}(s_{n}|y_{1:n})=\sum_{i=1}^{N}\pi_{n}^{\left(i\right)}\delta_{s_{n}^{\left(i\right)}}(\text{d}s_{n})$. More details can be found in Blake & Isard (1998), Isard & Blake (1998), Doucet *et al*. (2001) and Shen *et al*. (2006).

### 2.4 Cell tracking

**Figure 2:**
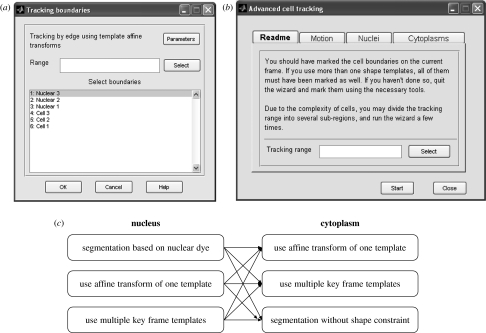
Screen shots illustrating some of the choices available to the user for tracking cells within CellTracker. (*a*) Interface for tracking cell boundaries. (*b*) Wizard for tracking both nuclear and cell boundaries. (*c*) A chart to illustrate the combinations available as algorithm options in the wizard.

The term ‘cell tracking’ here means tracking of both nuclear and cytoplasmic boundaries for each cell. The first step in tracking is initialization. Initialization of the cell boundaries may be generated by the cell detection algorithm described earlier (or may be done manually). The tracking parameters vary according to the combination of tracking algorithms mentioned earlier. A chart of possible combinations available via the interface is given in figure 2. CellTracker provides a wizard to guide users to a reasonable combination. Note that CellTracker provides tracking at the boundary level, and the software provides an interface for tracking selected boundaries using a variety of methods.

### 2.5 Import and export

In CellTracker, a time-lapse image series is imported for tracking. Currently, it supports Carl Zeiss LSM, tiff and Matlab mat files. The LSM file is essentially an extension of the TIFF multiple image stack file format, and it thereby accommodates any number of user-defined channels, e.g. those based on the fluorescence at different wavelengths. CellTracker may export a variety of calculated image data and a video of selected snapshots. The CellTracker software produces cell boundaries for each frame. One can also export cell properties, such as the nuclear and cytoplasmic average intensities, into Microsoft Excel and to an XML file. The XML file, whose schema we give on the website http://dbkgroup.org/celltracker, includes the tracking methods and cell boundaries for each frame, which can be exported into our information management system. With cell boundaries and image data available, users may calculate other properties without using the CellTracker.

## 3. Experimental

SK-N-AS cells (Nelson *et al*. 2004) were plated in 35 mm glass-bottomed tissue-culture dishes (Iwaki, Japan) containing 3 ml of minimal essential medium with Earle's salts (Gibco, UK) plus 10% (v/v) foetal bovine serum (Harlan Seralab, UK) and 1% non-essential amino acids (Gibco, UK). Twenty-four hours post-plating, the cells were co-transfected with p65-DsRed and pEGFP-N1 expression vectors, which produced a red fluorescent p65 (relA) fusion protein and enhanced green fluorescent protein. Twenty-four hours post-transfection, the cells were stimulated with TNF-α and imaged by confocal laser scanning microscopy. The microscopy was carried out using a Zeiss LSM510 confocal microscope equipped with a humidified CO_2_ incubator (37 °C, 5% CO_2_) and a 40×immersion objective (numerical aperture=1.3). Excitation of enhanced green fluorescent protein was performed using an argon ion laser at 488 nm and the emitted light was detected that was reflected through a 505–550 nm bandpass filter from a 545 nm dichroic mirror. DsRed fluorescence was excited using a green helium–neon laser (543 nm) and was detected through a 545 nm dichroic mirror and a 560 nm longpass filter. Data capture and manual analysis were carried out with LSM510 v. 3.2 software (Zeiss, Germany). The mean fluorescence intensities per pixel of DsRed fusion proteins were calculated for each time point for both nuclei and cytoplasm using the physiology option in the LSM510 v. 3.2 software, from which the nuclear to cytoplasmic (Nuc : Cyto) fluorescence intensity ratios were calculated and plotted using Microsoft Excel.

The automatic tracking has been tested on various computers. The running time given in this paper was based on the results using a portable computer with 1.7 GHz Intel CPU and 1 Gb RAM.

## 4. Results and discussion

**Figure 3:**
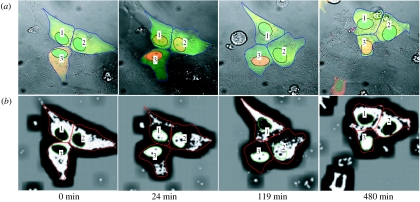
Image sequence of NF-κB at the times stated (following the addition TNF-α). (*a*) Original images. (*b*) Images of the DsRed channel after pre-processing.

**Figure 4:**
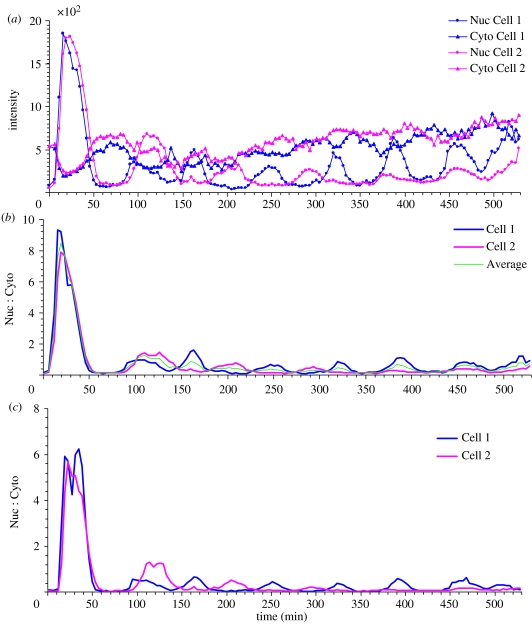
Cell tracking results. (*a*) Average fluorescent intensities of nuclei and cytoplasm. (*b*) Nuc : Cyto ratio profiles obtained by CellTracker. (*c*) Nuc : Cyto ratio profiles obtained by a biologist.

Figure 3*a* shows four typical frames recorded in an NF-κB signalling experiment. The cells touch each other, and the cell positions and boundaries change over time. It is therefore very time consuming to draw the cell boundaries manually. In this example, no nuclear or cytoplasmic dyes were added to aid tracking. However, the signal from the p65-DsRed fusion protein often provides a clear contrast between the nucleus and cytoplasm of the cells, and can be used for tracking via the image edge features. The fluorescent intensities between cells may vary considerably between cells and over time. This makes it difficult to find edge features using fixed parameters. In this example, we first smooth the images using a median filter of size 5, and normalize the images using a local average and its standard deviation. The normalized images of the p65-DsRed channel are shown in figure 3*b*. The cell fluorescent intensities after normalization are then at the similar levels. Note that the shape of the nuclei approximates to ellipsoids, which may be used as a template. On the other hand, the shape of the cytoplasm is both irregular and highly variable, and it is difficult to define a limited number of shape templates. Using the nuclei as seeds, the segmentation method based on Voronoi can be applied to estimate the cellular boundaries. The results (see figure 3*b* and the electronic supplementary material) show how CellTracker is able to capture the motion of cells and their size variations. Figure 4*a* shows the average fluorescent intensity of nuclei and cytoplasm measured using CellTracker, while figure 4*b* gives the equivalent nucleus : cytoplasm (Nuc : Cyto) ratio. There is a clear oscillation pattern in the profiles (as seen previously, e.g. Nelson *et al*. 2004). The results obtained by the CellTracker agree with these profiles obtained using manual analysis (figure 4*c*), but were obtained over 20 times more quickly (approx. 5 versus 120 min). Obviously, due to human fatigue, this ratio will grow substantially, and the automated data outstrip the manual analyses in terms of quality, with increases in the number of images analysed. (The gene expression levels of cell 3 are not shown here because the image intensities saturated at some time points.)

In high-content image analysis, it is often the case that populations of cells (often dead or fixed) are analysed as a whole. The cells in this example show clearly that the location of signalling proteins can oscillate in each cell but that they are out of phase with each other when comparing different cells. This causes them to be damped out if they are analysed at the level of the population (cf. Davey & Kell 1996; Nelson *et al*. 2002*a*, 2004). The dotted line in figure 4*b* shows the average Nuc : Cyto ratio of cells 1 and 2. Its oscillation pattern is clearly quite different quantitatively from those of the individual cells, and as the properties of more cells are time averaged, this pattern becomes increasingly blurred. Therefore, it is impossible to establish the mechanisms of signalling that occur in individual cells, and effect their comparison with systems biology-type models, using results from a population. Indeed, based on single-cell analyses, it was reported that the functional consequences of NF-κB signalling may in fact depend not only on the signal amplitude, but also on the number, period and frequency of these oscillations, i.e. their detailed dynamics (Nelson *et al*. 2004; Kell 2006), underscoring the importance of single-cell measurements.

The above paragraphs have summarized many of the chief properties of CellTracker, but many other features are available and are described in full in the software itself and its manual. To this end, we have made CellTracker available for download via the URL http://dbkgroup.org/celltracker/, together with a variety of files illustrating various features including multiparameter analyses. We believe that it has the most comprehensive facilities available for live-cell tracking, and trust that it may prove useful to the high-content screening community.

## Figures and Tables

**Table 1:** Fields used to describe a boundary.

field	function
ID	a unique integer as the boundary
data	a two-column matrix with *x*, *y* coordinates
control point	spline control point to control data
tag	boundary type
label	boundary name
label position	one may label a boundary at a couple of positions
children	sub-boundary IDs
parent	parent boundary IDs

## Appendix A. Colour-based tracking

The colour distribution $p_{y}={\left\{p_{y}^{u}\right\}}_{u=1,\ldots ,m}$ of a region *R* at location *y* is calculated as

(A1) $$p_{y}^{u}\propto \sum_{x_{i}\in R}k\left(\frac{\left\|y-x_{i}\right\|}{a}\right)\delta [h(x_{i})-u],$$

where *δ* is the Kronecker delta function and *h*(*x*_*i*_) assigns one of the *m*-bins of the histogram to a given colour at location *x*_*i*_. The variable *a* provides invariance against scaling of the region. The colour distribution is normalized to ensure that $\sum_{u=1}^{m}p_{y}^{u}=1$. A popular measure for assessing the difference between two distributions is the Bhattacharyya coefficient (Nummiaro *et al*. 2003). Considering discrete densities, such as two-colour histograms $p={\left\{p^{u}\right\}}_{u=1,\ldots ,m}$ and $q={\left\{q^{u}\right\}}_{u=1,\ldots ,m}$, the coefficient is defined as

(A2) $$\rho (p,q)=\sum_{u=1}^{m}\sqrt{p^{u}q^{u}}.$$

The larger the *ρ* is, the more similar are the distributions. For two identical histograms, we obtain *ρ*=1, indicating a perfect match. The distance between two distributions is measured by Bhattacharyya distance

(A3) $$d=\sqrt{1-\rho (p,q)}.$$

The quality of the colour-based particle filter can be influenced by the illumination conditions, the visual angle and the camera parameters. To overcome this, we update the target model when image observations are slowly changing. By discarding image outliers where the object is occluded or too noisy, the tracker can be protected against updating the model when the object has been lost. Thus, we use the update rule $\pi_{E[s]}>\pi_{\text{T}}$ where $\pi_{E[s]}$ is the observation probability in terms of the mean state *E*[*s*] and *π*_T_ is a threshold. The update of the target model is implemented by the equation

(A4) $$q_{t}^{u}=(1-\alpha )q_{t-1}^{u}+ap_{E[s_{t}]}^{u},$$

for each bin *u*, where *α* weighs the contribution of the mean state histogram $p_{E[s_{t}]}$ to the target model $q_{t-1}$. Thus, we invoke a ‘forgetting’ process, in the sense that the contribution of a specific frame decreases exponentially the further it lies in the past.

## Supplementary Material

Automated tracking of cells in an image sequence SK-N-AS cells, incorporating NK-κB tagged with DsRed, were stimulated with TNFα and imaged by confocal laser scanning microscopy. The overall period of observation is 480 min.
